# Judgment Bias During Gestation in Domestic Pigs

**DOI:** 10.3389/fvets.2022.881101

**Published:** 2022-05-12

**Authors:** Emily V. Bushby, Sheena C. Cotter, Anna Wilkinson, Mary Friel, Lisa M. Collins

**Affiliations:** ^1^School of Life Sciences, University of Lincoln, Lincoln, United Kingdom; ^2^School of Biology, Faculty of Biological Sciences, University of Leeds, Leeds, United Kingdom

**Keywords:** pregnancy, gestation, cognitive bias, affective state, information processing, pig

## Abstract

In humans and rats, changes in affect are known to occur during pregnancy, however it is unknown how gestation may influence mood in other non-human mammals. This study assessed changes in pigs' judgment bias as a measure of affective state throughout gestation. Pigs were trained to complete a spatial judgment bias task with reference to positive and negative locations. We tested gilts before mating, and during early and late gestation, by assessing their responses to ambiguous probe locations. Pigs responded increasingly negatively to ambiguous probes as gestation progressed and there were consistent inter-individual differences in baseline optimism. This suggests that the pigs' affective state may be altered during gestation, although as a non-pregnant control group was not tested, an effect of learning cannot be ruled out. These results suggest that judgment bias is altered during gestation in domestic pigs, consequently raising novel welfare considerations for captive multiparous species.

## Background

Research investigating the links between pregnancy, affect and cognition is most often carried out with a human-centric focus with studies typically using case studies and cohorts. In humans, changes in affective state during pregnancy are common and alterations in levels of anxiety, depression and cognitive ability have been demonstrated in humans and rodents (1–3). These changes are often linked to the large and rapid hormone fluctuations that occur during the gestational period (4, 5). Where human subjects cannot be used, rodent models are often employed to experimentally investigate how factors such as diet, enrichment or stress can influence behavior during gestation (6–8). To infer anxiety and depressive-like behaviors, lab-based behavioral tests, such as a forced swim or open-field test are often used (9). These studies are conducted under laboratory conditions and are generally aimed at modeling human gestation, rather than investigating how gestation may impact on the rodent itself. Results from both human and rodent studies are varied, however most show that affective state is altered throughout gestation [for review see (2)] and it is clear that pregnancy impacts maternal affective state.

Understanding an animals' affective state better enables us to understand their subjective experience, both positive and negative, and is a key component of animal welfare (10). Affective state can influence and alter cognitive processes, such as judgment (11, 12), which may then be used to infer and understand an animals' affective state. Cognitive bias or judgment bias is the influence of affect on information processing, with more content individuals likely to make positive assumptions about ambiguous stimuli, and vice versa (13). Judgment bias tests have been used to assess changes in affective state in a range of species, including pigs, dogs, honeybees and European starlings (14–17). Research typically focuses on the impact of external stimuli on judgment bias; this is likely to act via alteration to the internal, physiological environment ultimately resulting in changes in behavior and judgment bias (11, 18, 19). As such, we would expect internal stimuli, such as physiological changes, would also impact judgment bias directly even in the absence of external influences. Pregnancy is one of the biggest physiological changes a mammal may experience, involving major hormonal and cognitive adjustments (20, 21), yet little is known of how information processing and affective state may change in relation to gestation in animals.

The domestic pig *(Sus scrofa domesticus)* has been used as a human model in a wide range of medical research such as infectious disease (22), nutritional (23) and neurological studies (24). Pigs allow for longer lifespan studies and are more anatomically and physiologically similar to humans than other laboratory species, such as rodents (25, 26). More commonly, pigs are farmed around the globe for meat production. Modern intensive farming systems have been designed to produce food as quickly and cost efficiently as possible, and research is continually ongoing to understand how animal welfare can be optimized within these systems. Despite many studies on the behavioral and welfare needs of sows during gestation (27–30), only two studies used a specific judgment bias task to assess affective state in gestating sows. These studies focused on using judgment bias as a welfare indicator in gestating sows however, did not investigate how gestation itself influenced judgment bias (31, 32). More recently another study showed that gestating gilts that were classified as “friendly” visited an electronic sow feeder more often than individuals that were classified as “fearful” (33). The authors hypothesized that this feeding behavior may be similar to a judgment bias task and that the friendly individuals may have been more optimistic. However, again this study did not investigate how gestation itself influenced judgment bias.

We investigated how gestation may alter judgment, and therefore affective state, in domestic pigs. We compared within-individual affective state, as measured by a spatial judgment bias test, before mating, and during early and late gestation. We hypothesized that within-individual judgment bias would be more pessimistic during gestation than prior to mating, leading to an increase in latency to approach ambiguous cues throughout gestation. This is the first study to our knowledge to investigate the possible impact of gestation on judgment bias in domestic pigs.

## Methods and Materials

This work was carried out between July and October 2015 (replicate one) and between January and July 2017 (replicate two) on a pig farm in the UK.

### Animal Housing and Husbandry

20 gilts (primiparous female pigs; *N* = 10 for each replicate) were selected based on age and time until first mating. Using gilts allowed for training time before gestation, as there is limited time between pregnancies once a sow has begun breeding. The average age of all 20 pigs on day one of training was 241.7 (SD: 15.93) days. Replicate two contained one Duroc and three Landrace pigs, the breed of all other individuals was Large White. Pigs were housed in pens of five or six animals, each pen (4.67 × 5.35 m) contained a sheltered sleeping area with straw bedding (2.70 × 4.67 m) and a run partially exposed to outdoor elements, such as wind and natural light (2.65 × 4.67 m). A standard lactating sow ration was fed once a day before mating and throughout gestation; there was continuous access to water and natural lighting. During the course of the study the animals remained within the same groups and pens to keep the external environment as controlled as possible throughout. The study pigs were able to interact with pigs in the pen next door via the gate and animals in the Neighboring pens may have been moved/changed. Due to involvement in a separate study, replicate one pigs received Regumate^®^ (containing a steroidal progestin) orally with feed 23 days before planned estrus to allow for Synchronized farrowing. As of June 2020, no previous research was found investigating possible effects of Regumate^®^ on affective state or behavior of pigs. Due to this research taking place on a working farm, it was not possible to test a separate non-pregnant control group and each pig was used as its own control.

### Judgement Bias

**Figure 1:**
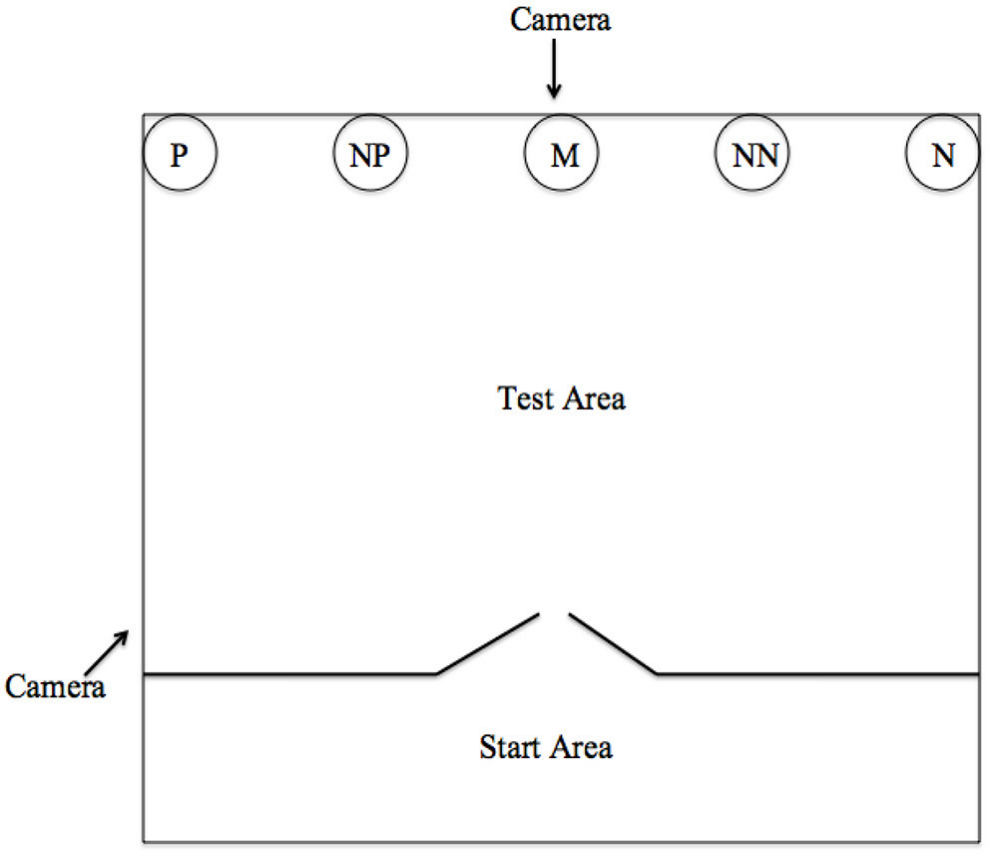
Experimental set up for the judgement bias test with positive (P), near positive (NP), middle (M), near negative (NN), and negative (N) locations. The figure shows the locations for an individual trained to expect a positive reward in the left corner and to avoid the right. Only one bowl was present in one location at a time.

The training and testing area (Figure 1) used comprised of a testing room (3.72 × 5.26 m) and a starting room (3.72 × 1.79 m). All pigs were habituated to the test area in groups for two to three sessions, and then individually for a maximum of seven sessions to habituate the pigs to eating from a bowl which was initially placed in the center of the test area. Following this, individuals were trained to associate the bowl in two opposite corner locations with a positive (*P*) and a negative (*N*) outcome. When in the P location, the bowl contained a small amount of chocolate raisins (replicate 1) or sugar-coated chocolates (replicate 2) and when it was in the N location, the bowl contained unpalatable food (bitter tasting coffee beans) to discourage the pigs from approaching this location. The pigs were trained to discriminate between these reference locations by first only receiving positive trials and then later interspersing negative trials. Latency to reach the bowl was recorded using video cameras and was then used as a metric to assess whether each individual had learned the discrimination. Each trial was 30 s in duration. Correct responses were recorded when the subject approached and touched their nose to the bowl during the positive (P) trials; during negative (N) trials, a correct response was recorded when the individual did not approach the bowl within 30 s. The location of P and N was counterbalanced across individuals. For both replicates a criterion of 70% correct responses in the last 20 trials was required before moving onto the testing phase. Per individual, forty-four training trials were conducted during replicate one and sixty-two for replicate two. Replicate two required more training trials due to the pigs being slower to differentiate between the positive and negative locations, though had a higher rate of meeting the criterion by the end of training. Five pigs from replicate one failed to meet this criterion and were removed from the study. Two pigs from replicate 2 did not meet this criterion. The analysis represents only those 13 that met the learning criterion.

Each testing session comprised two sets of nine trials carried out on the same day, involving five different bowl locations; the three intermediate ambiguous probes: near positive (NP), middle (M) and near negative (NN), interspersed with P and N reference locations (e.g. P, N, M, P, N, NP, P, N, NN). Only one bowl was in the arena during each trial. The ambiguous probes were placed in predetermined equidistant positions (0.74 m) and were not reinforced (i.e., they were left empty). They were presented in a pseudo-randomized order and interspersed among training trials. All “during gestation” testing sessions were preceded by five “reminder” training trials the day before testing. Each pig was tested three times: before gestation (1–2 weeks before mating); early gestation (4 weeks after mating); and late gestation (10–11 weeks after mating). One pig in replicate two was not tested before gestation and was only tested in the early and late test phases.

### Statistical Analysis

All data were analyzed in R version 3.4.1 using general linear mixed effects models with the *lmer* function in the package *lme4* (34). The response variable was natural logged to ensure that the residuals conformed to the assumptions of normality. To test the effects of gestation time on judgment bias, the response variable was *log time taken to approach* the presented probes; fixed explanatory effects were *probe location*, coded as a continuous variable from positive (1) to negative (5) with ambiguous locations at points 2, 3, and 4; and *gestation time* coded as a factor with three levels (pre, early and late gestation). *Probe location squared* was included as initial data exploration suggested curvature in the fits. Interactions between *gestation time* and *probe location* and *probe location squared* were also included.

To find the most appropriate structure for the random model, we compared eight models: two intercept only models and six combinations of random intercept and slope models such that random intercepts were fitted for each pig at each experimental timepoint (or for each pig independent of experimental replicate), with variation allowed between gestation times and the shape of the curve was allowed to vary between pigs (Table 1).

**Table 1:** Statistical model details.

**Model**	**Random slope**	**Random intercept**
1	1	Gestation time: Pig ID
2	1	Replicate/Gestation time: Pig ID
3	Location	Gestation time: Pig ID
4	Location	Replicate/Gestation time: Pig ID
5	Location^2^	Gestation time: Pig ID
6	Location^2^	Replicate/Gestation time: Pig ID
7	Location + location^2^	Gestation time: Pig ID
8	Location + location^2^	Replicate/Gestation time: Pig ID

*Random models with fixed slopes (models 1 and 2) or slopes allowed to vary across probe location (models 3–8), with experimental replicate included (models 2, 4, 6, and 8) or not (models 1, 3, 5, and 7)*.

The Akaike Information Criteria (AIC) values for all models were compared using the *model.sel* function in the *MuMIn* package (35). In each case the residuals of the final minimal model were visually assessed for deviations from normality. For the final models, predicted fits were produced using the *predict* function in base *R*. *R*^2^ values for each model were calculated using the *r.squaredGLMM* function in the *MuMIn* package (35). For every model, the general pattern of results was robust, with the different random models only affecting the predictions very slightly. The best model is reported in the main text, and the corresponding figure for the other model where AIC comparison had delta <2 is reported as supplementary information.

## Results

### Judgment Bias

**Figure 2:**
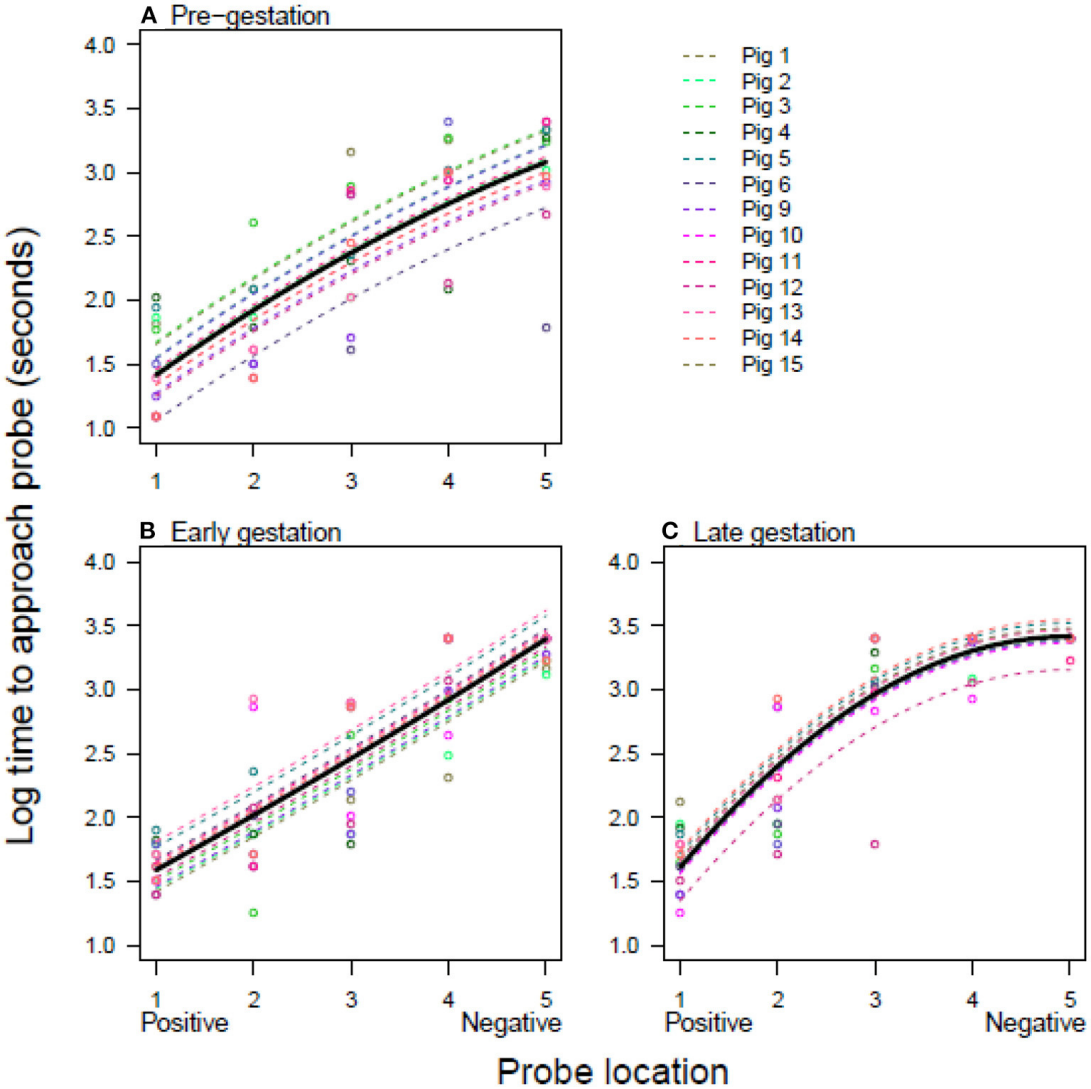
The time to approach each location at three stages of gestation. Log time taken to approach each location for pigs at three different stages of the pig's 16-week gestational period; **(A)** pre-gestation, **(B)** early gestation (5 weeks), and **(C)** late gestation (10–11 weeks). The open circles are raw data points and the lines are model predictions from the minimal adequate model fixed to the level of experimental replicate 1. Results from model 1 are shown, where the intercept is allowed to vary for each pig at each gestation time. Pigs 1–5 are from replicate 1 and pigs 6–15 are from replicate 2.

The pigs' responses to ambiguous locations in the judgement bias test changed throughout gestation (Tables 2, 3; Figure 2). Pigs consistently approached the positive probe quickly and the negative probe slowly (or not at all), getting generally slower during gestation (Figure 2). However, whilst the mean speed of approach was fairly linear between positive and negative pre- and early gestation (Figures 2A,B), by late gestation, pigs showed a shift toward pessimism, such that the positive probe continued to be approached quickly but ambiguous probes were approached more slowly (Figure 2C).

**Table 2:** Table of candidate LMERs.

**Model**	***df***	**AIC_**c**_**	**Δ AIC_**c**_**	**w**	***r*^2^ (*F* only)**	***r*^2^ (*F* + *R*)**
1	12	215.2	0.00	0.580	0.751	0.805
2	11	216.9	1.72	0.245	0.748	0.806
3	16	219.7	4.50	0.061	0.751	0.805
5	13	219.8	4.57	0.059	0.751	0.805
7	13	221.2	6.01	0.029	0.750	0.818
4	22	222.2	7.02	0.017	0.738	0.810
6	16	224.1	8.89	0.007	0.740	0.817
8	16	227.5	12.35	0.001	0.729	0.833

Table of candidate LMERs explaining time to approach the probe in relation to the interaction between the location of the presented probe and the gestation time for pigs that reached the 70% learning criterion only (n = 13). Each model retained all fixed terms (Location*Gestation time+Location^2*^Gestation time) with only the random model varying. Model corresponds to the random model listed in Table 1, AIC_c_, corrected Akaike Information Criteria values; Δ AIC_c_, difference in AIC_c_ values between the best model (lowest AIC_c_) and the given model; w, Akaike weights; r^2^ (F only), r^2^ for the fixed model only, r^2^ (F + R) r^2^ for the fixed plus random model.

**Table 3:** Results of the best supported statistical models.

	**Model 1**			**Model 2**		
**Term**	**DF**	***F***	***P***	**DF**	***F***	***P***
Location	1, 141	62.96	<0.001	1, 141	62.96	<0.001
Gestation time	2, 168	2.03	0.134	2, 167	2.04	0.133
Location^2^	1, 141	9.57	0.002	1, 141	9.57	0.002
Location: gestation time	2, 141	6.07	0.003	2, 141	6.07	0.003
Location^2^: gestation time	2, 141	6.16	0.003	2, 141	6.16	0.003

*Minimum adequate linear mixed effects model for the effects of probe location and gestation time on the time taken for pigs to approach the probe under testing, for pigs that reached the 70% learning criteria only (n = 13). The results equate to the best supported random models*.

All models retained all interactions and gave qualitatively similar results. The best model was model 1, where the intercept was allowed to vary for each pig at each gestation time (Table 1). However, the result for model 2, where the intercept was allowed to vary for each pig at each gestation time, within each replicate, was equally well supported (delta AIC <2; Table 2, Supplementary Figure 1).

## Discussion

In livestock species, judgment bias tasks are typically used to assess the impact of external factors, for example environmental enrichment (36) or stocking density (37). However, internal factors, such as the large physiological changes associated with gestation, also have the potential to influence affective state and therefore judgment bias. The aim of this study was to assess judgment bias in domestic pigs throughout gestation. It was hypothesized that the gilts would be more pessimistic during gestation than prior to mating, as indicated by an increase in latency to approach the ambiguous cues. Our results suggest this to be the case, with the gilts taking longer to approach the ambiguous locations in the later stage of gestation than before mating which indicates that judgment bias changed as gestation progressed. This was most apparent at the middle and most ambiguous location (Figure 2) and suggests the pigs were more pessimistic during the late gestational stage. Crucially, the latency to reach the positive location did not vary markedly throughout gestation, highlighting that changes, such as impaired locomotion or an increase weight, did not affect the gilts' response latencies to the other four locations (Figure 2). This also shows that the gilts were highly motivated by the reward, even though they were not feed-restricted. Thus, these results suggest increased pessimism during the late stage of gestation, despite the fact that the immediate external environment remained constant. This may infer that, alongside external factors, internally-driven factors can also influence judgment bias and affective state in domestic pigs. Although this result should be interpreted in light of the pigs being their own control and no separate control group being tested.

In this study, a spatial go/no-go judgment bias test was used as this type of task has been successfully used with pigs previously (14, 31, 32, 36). Previous judgment bias studies with livestock species have shown that a change of bias can occur in response to a change in external factors, such as enrichment (36) or handling (38). Recent studies by Horback and Parsons (31, 32) also used a spatial go/no-go task and found that group housed gestating sows displayed both positive and negative biases despite having the same external conditions. Interestingly, the sows' behavioral traits influenced judgment bias however, these studies were not specifically focusing on the effect of gestation on judgment bias, and therefore it is unclear if the stage of gestation may also play a role in these bias differences. The possibility that pigs' judgment bias may change from a positive to a more negative state during the late stage of gestation suggests that the pigs' welfare needs may change too. This highlights the importance of considering the impact of large physiological changes, such as gestation, on animal welfare. This study may have implications not only for the welfare of farmed animals that experience gestation, but also for research into affective state during gestation in other captive multiparous mammalian species, including how this may impact cumulatively across the life course on their health and welfare. For example, in humans, multiparous women appear to be more at risk and have a different pattern of anxious or depressive symptoms compared to primiparous women (39, 40). In humans, hormone fluctuations and other physiological changes throughout pregnancy are often correlated with changes in mood and affective state (4, 5). Pigs are frequently used as models for humans in medical and pharmaceutical studies (22, 23, 41, 42), so it is possible that a change in affective state during gestation may be caused by comparative mechanisms, however, further research is required to validate this.

Alongside this interesting result, there are some limitations to take into consideration. Previous studies have shown that multiple testing time points can result in an increase in pessimistic responses (43, 44) and this increase in latencies during the later testing phases is similar to what was found in this study. As it was not possible to test a non-pregnant control group, this effect of learning cannot be ruled out. However, the effects of gestation represent a plausible driver for the changes in affect we report as previous research in rodents and humans has shown that mood and affective state can vary throughout gestation (1–3), with negative mood more likely to be present during the first and third trimester in humans (45, 46). Future studies should consider the role of learning by including a non-gestating control group, and whether ambiguous trial locations should be rewarded or un-rewarded (47). There were also some differences between replicates, such as one replicate receiving Regumate^®^, and different rewards being used. Despite this, the effect of replicate on the data was marginal, showing that the change in judgment bias over the course of gestation was robust and not influenced by these differences between replicates.

In conclusion, this study suggests that judgment bias in farmed domestic pigs may change with stage of gestation, inferring that internally driven stimuli may directly affect judgment bias without external influence. This study raises novel welfare considerations for captive primiparous, and possibly multiparous, species and provides a basis for future research into the effect of gestation on judgment bias in non-human animals.

## Data Availability Statement

The data and R code is available from the Open Science Framework database: https://osf.io/32wgy/.

## Ethics Statement

The University of Lincoln, College of Science Ethics Committee approved this study (COSREC189; COSREC262). The animal owners provided written informed consent for participation of their animals in this study.

## Author Contributions

EB carried out data collection. LC conceived of the study. SC carried out statistical analysis. All authors assisted with study design and coordination, drafting the final manuscript, and gave final approval for publication.

## Funding

LC and MF were funded by the Biotechnology and Biological Sciences Research Council Grant BB/K002554/2.

## Conflict of Interest

The authors declare that the research was conducted in the absence of any commercial or financial relationships that could be construed as a potential conflict of interest.

## Publisher's Note

All claims expressed in this article are solely those of the authors and do not necessarily represent those of their affiliated organizations, or those of the publisher, the editors and the reviewers. Any product that may be evaluated in this article, or claim that may be made by its manufacturer, is not guaranteed or endorsed by the publisher.
