# Supplementary material for: Judgment Bias During Gestation in Domestic Pigs
Source: Front Vet Sci. 2022 May 12;9:881101. doi: 10.3389/fvets.2022.881101 (PMC9133791; doi:10.3389/fvets.2022.881101)
Supplement: Supplementary file 1 [file Table_1.DOCX]

## Supplementary Material

## Figure S1: The latencies for all 13 pigs to approach each location during three stages of gestation. Log time taken to approach each location for pigs at three different stages of the pig’s 16-week gestational period; a) pre-gestation, b) early gestation (5 weeks) and c) late gestation (11 weeks). The open circles are raw data points and the lines are model predictions from the minimal adequate model fixed to the level of experimental replicate 1. Results from model 2 are shown, where the intercept is allowed to vary for each pig at each gestation time, within each replicate. Only the 13 pigs that had >70% correct responses during the learning phase are included.
